# Spatiotemporal Distribution and Assemblages of Planktonic Fungi in the Coastal Waters of the Bohai Sea

**DOI:** 10.3389/fmicb.2018.00584

**Published:** 2018-03-28

**Authors:** Yaqiong Wang, Biswarup Sen, Yaodong He, Ningdong Xie, Guangyi Wang

**Affiliations:** ^1^Center for Marine Environmental Ecology, School of Environment Science and Engineering, Tianjin University, Tianjin, China; ^2^School of Ecology, Environment and Resources, Qinghai University for Nationalities, Xining, China; ^3^Duke Marine Laboratory, Nicholas School of the Environment, Duke University, Durham, NC, United States; ^4^Key Laboratory of Systems Bioengineering (Ministry of Education), Tianjin University, Tianjin, China

**Keywords:** marine ecosystem, water column, abundance, diversity, quantitative PCR, high-throughput sequencing

## Abstract

Fungi play a critical role in the nutrient cycling and ecological function in terrestrial and freshwater ecosystems. Yet, many ecological aspects of their counterparts in coastal ecosystems remain largely elusive. Using high-throughput sequencing, quantitative PCR, and environmental data analyses, we studied the spatiotemporal changes in the abundance and diversity of planktonic fungi and their abiotic and biotic interactions in the coastal waters of three transects along the Bohai Sea. A total of 4362 ITS OTUs were identified and more than 60% of which were unclassified Fungi. Of the classified OTUs three major fungal phyla, Ascomycota, Basidiomycota, and Chytridiomycota were predominant with episodic low dominance phyla Cryptomycota and Mucoromycota (Mortierellales). The estimated average Fungi-specific 18S rRNA gene qPCR abundances varied within 4.28 × 10^6^ and 1.13 × 10^7^copies/L with significantly (*P* < 0.05) different abundances among the transects suggesting potential influence of the different riverine inputs. The spatiotemporal changes in the OTU abundance of Ascomycota and Basidiomycota phyla coincided significantly (*P* < 0.05) with nutrients traced to riverine inputs and phytoplankton detritus. Among the eight major fungal orders, the abundance of Hypocreales varied significantly (*P* < 0.01) across months while Capnodiales, Pleosporales, Eurotiales, and Sporidiobolales varied significantly (*P* < 0.05) across transects. In addition, our results likely suggest a tripartite interaction model for the association within members of Cryptomycota (hyperparasites), Chytridiomycota (both parasites and saprotrophs), and phytoplankton in the coastal waters. The fungal network featured several hubs and keystone OTUs besides the display of cooperative and competitive relationship within OTUs. These results support the notion that planktonic fungi, hitherto mostly undescribed, play diverse ecological roles in marine habitats and further outline niche processes, tripartite and co-occurrence interaction as the major drivers of their community structure and spatiotemporal distribution in the coastal water column.

## Introduction

Microbial plankton governs the ecological function of the marine ecosystem by sustaining food webs and regulating global biogeochemical cycles (Rousk and Bengtson, 2014; Worden et al., 2015). Recently, molecular approaches have revealed the vast and complex diversity of microbial plankton groups (e.g., bacterioplankton and protist) and their intrinsic relationship with a wide range of environmental drivers (Caporaso et al., 2012; Logares et al., 2014; de Vargas et al., 2015). Fungi have long been known to be a key component of biosphere involved in a wide range of biogeochemical cycles, ecological functions across disparate terrestrial environments (Christensen, 1989; Carlile et al., 2001; Pang and Mitchell, 2005; Fischer et al., 2006; Gulis et al., 2006), and natural product research (Pang et al., 2016). Planktonic fungi, which include morphologically diverse zoosporic fungi, free-living filamentous and yeast forms or parasites of the other planktons (Richards et al., 2012; Wang et al., 2012), are known for several decades about their existence in ocean waters. The function of their planktonic forms in marine ecosystems only has been recognized recently (Gao et al., 2010; Wang et al., 2014; Taylor and Cunliffe, 2016), but evidence to support their function is still lacking.

As one of the most dynamic ecosystems, coastal waters are generally characterized with a high biodiversity and high primary production (Danovaro and Pusceddu, 2007). Planktonic fungi are considered to decompose detrital organic matter or phytoplankton-derived organic matters and utilize dissolved organic carbon with a noticeable contribution to secondary production in the coastal marine ecosystems (Kimura and Naganuma, 2001; Kimura et al., 2001; Gao et al., 2010; Gutiérrez et al., 2011). Nevertheless, these ecological roles are largely proposed based on the comparison with their counterparts in terrestrial and freshwater ecosystems (Richards et al., 2012; Taylor and Cunliffe, 2016). The recent discovery of fungal mycelia (i.e., metabolically active forms of fungi) to exist as individual filaments or aggregates in coastal ocean waters (Gutiérrez et al., 2010; Li Q. et al., 2016) support the hypothesis that planktonic fungi are active. Furthermore, high fungal biomass can be comparable to that of planktonic prokaryotes and have been noticed to relate with an increase in phytoplankton biomass and in extracellular enzymatic hydrolysis in coastal waters (Gutiérrez et al., 2011). The vertical distribution patterns (e.g., diversity and species richness) of planktonic fungi have also been consistent with those of phytoplankton (Gao et al., 2010; Gutiérrez et al., 2011; Wang et al., 2014). The above findings clearly suggest the important role of planktonic fungi in the coastal waters, and like other heterotrophic plankton groups, they are tightly linked with primary production and organic matter.

An enormous fungal diversity has been reported from terrestrial environments (Hawksworth, 2001; O’Brien et al., 2005; Hibbett et al., 2007; Mueller et al., 2007). Relative to other marine plankton groups and their counterparts in terrestrial and freshwater ecosystems, the diversity of marine planktonic fungi and their response to environmental gradients remain quite limited (Fell and Newell, 1998; Raghukumar, 2005; Gulis et al., 2006; Gessner et al., 2007; Gao et al., 2010; Wang et al., 2012, 2014). Molecular-based approaches have revealed high diversity of planktonic fungi with novel lineages in the coastal water columns, displaying interesting temporal and spatial (lateral and vertical) variations (Gao et al., 2010). This includes the higher diversity and a greater fungal abundance in the surface and coastal waters than in open-ocean and deep water samples (Gao et al., 2010). In addition, the diversity patterns of planktonic fungi have been related to major phytoplankton taxa and various nutrients in the oceanic waters (Wang et al., 2014). Time-series assessments have revealed several dominant planktonic fungi groups and their interesting relationship with environmental variables, including nitrogen availability and temperature at Coastal Station L4 in the Western English Channel, and suggested the significance of riverine inputs on their abundance and diversity in the coastal region (Taylor and Cunliffe, 2016). Together, previous studies show that planktonic fungi are molecularly diverse and that a variety of fungal phylotypes, regulated by nutrients, mediate multiple biological processes in the coastal waters.

In our previous studies, we reported novel lineages of planktonic fungi and their relationship with environmental variables in the Hawaiian coastal waters and the pelagic waters of the Pacific Warm Pool (Gao et al., 2010; Wang et al., 2014). In this study, we applied high-throughput sequencing and qPCR tools to investigate the diversity and abundance of planktonic fungi in the semi-closed shallow bay ecosystem on the coast of Bohai Sea influenced by different riverine inputs. The specific objective of the present study was to realize the local dominant planktonic fungal groups, their spatiotemporal dynamics and factors governing their distribution patterns.

## Materials and Methods

### Characteristics of Sampling Area and Sample Collection

**FIGURE 1:**
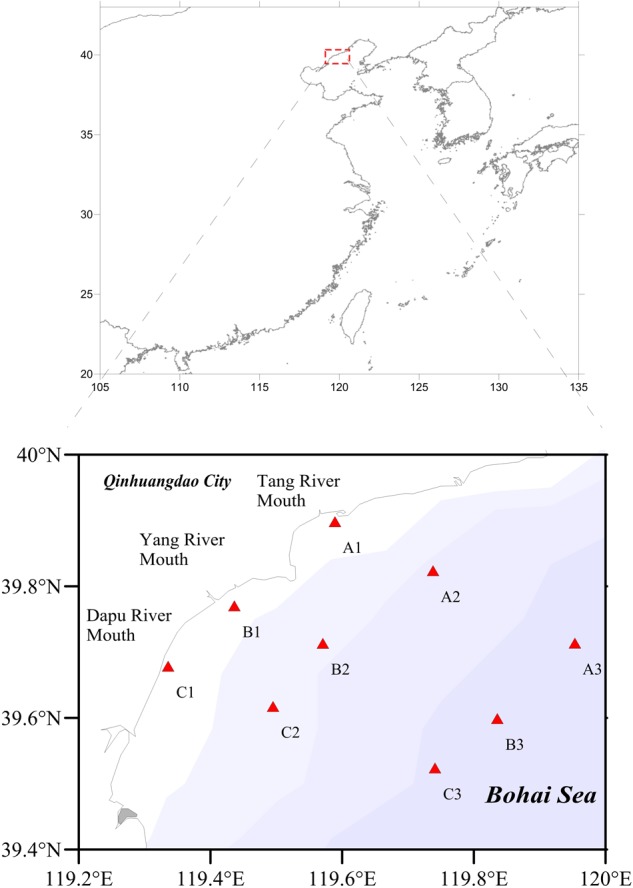
Map of sampling sites in Qinhuangdao coastal area.

The 125.4 km long coastline of the Qinhuangdao Coast stretches from the mouth of the Luan River to the city of Qinhuangdao on the northwest side of the Bohai Sea (**Figure 1**). Additionally, the Bohai Sea has been under the impacts of two branches of Yellow Sea warm circulation: one is a clockwise gyre toward Liaodong Bay, and the other is a counterclockwise gyre toward Bohai Bay (Wei et al., 2004). The semi-enclosed character of the Bohai Sea restricts water exchange and leads to rapid accumulation of pollutants in the environment (Zhang et al., 2009). In the Qinhuangdao coastal area of Bohai Sea, the brown tide outbreak occurs recurrently every April to August since 2009 (Zhang et al., 2012; Wang et al., 2015; Ma et al., 2016), thus, it remains a typical coastal ecosystem largely influenced by human and terrigenous inputs.

We set three sampling transects along the estuaries in Qinhuangdao coastal area: Section A, vertical shorelines from Tang River estuary extension to the sea; Section B, vertical shorelines from Yang River estuary extension to the sea; and Section C, vertical shorelines from Dapu River estuary extension to the sea. The gradient distribution consisted about 37 km long monitoring stations from nearshore to offshore and for each section, three sampling points were included (**Figure 1**). Seawater samples were collected with the standard sampling procedures from surface layer (0.5 m), bottom layer (the deepest depth of the station), and middle layer (5.0 m, when the depth of the station is more than 10 m) of nine locations (Station A1, Station A2, Station A3, Station B1, Station B2, Station B3, Station C1, Station C2, and Station C3) in the Qinhuangdao coastal area during November in 2014, April and July in 2015. Samples were designated ‘S’ (surface), ‘M’ (middle) and ‘B’ (bottom); ‘S11’ (November), ‘S4’ (April) and ‘S7’ (July), respectively in the following analysis. The information on sampling sites is shown in **Table 1**. We collected 2 L of water samples for each station, of which 500 mL was filtered through 0.22 μm polycarbonate filter membrane (Millipore, United States) for DNA extraction. The resulting filters for DNA extraction were stored at -80°C until further processing. The remaining seawater samples were used for environmental parameter determination. Water depth, temperature, salinity, dissolved oxygen (DO), and pH data were monitored at each sampling site using the corresponding sensors of YSI Pro Plus (Yellow Springs, OH 45387, United States). Water column samples were collected with polyvinyl chloride bottles. All water samples were stored in sterile containers at an *in situ* temperature and protected from direct sunlight until further processing in the laboratory.

**Table 1:** Information of sampling sites.

Stations	Longitude (E)	Latitude (N)	Sampling time	Layer	Depth (m)
A1	119°35′341″	39°53′899″	November, April, July	S	0.5
				B	8.0
A2	119°44′282″	39°49′415″	November, April, July	S	0.5
				M	5.0
				B	13.6
A3	119°57′209″	39°42′813″	April	S	0.5
				M	5.0
				B	22.5
B1	119°26′178″	39°46′211″	November, April, July	S	0.5
				B	7.1
B2	119°34′243″	39°42′802″	November, April, July	S	0.5
				M	5.0
				B	12.0
B3	119°50′135″	39°35′944″	April	S	0.5
				M	5.0
				B	22.0
C1	119°20′145″	39°40′711″	November, April, July	S	0.5
				B	5.0
C2	119°29′697″	39°37′052″	November, April, July	S	0.5
				M	5.0
				B	11.4
C3	119°44′462″	39°31′430″	April	S	0.5
				M	5.0
				B	20.4

### Determination of Environmental Parameters

Ammonium (NH_4_^+^), nitrate (NO_3_^-^), nitrite (NO_2_^-^), phosphate (PO_4_^3-^), and silicate (SiO_4_^3-^) were measured using QuAAtro39 Continuous Segmented Flow Analyzer (SEAL Analytical, Inc.). Chlorophyll *a*, total and dissolved phosphorus and nitrogen (TP, DP, and DN) were measured following the methods described elsewhere (Zhou et al., 2016). For DN and DP, seawater sample was filtered through a 0.45 μm Millipore filter (Millipore, United States). Dissolved inorganic and organic N (DIN and DON), dissolved inorganic and organic phosphorus (DIP and DOP), particulate total N and P (PP and PN) were calculated from the measured parameters described above. The nutrient concentrations in the samples are provided in Supplementary Table S1.

### DNA Extraction, PCR Amplification, and High-Throughput Sequencing

The total genomic DNA was extracted from the membrane filters using the E.N.Z.A.^TM^ Water DNA Kit (Omega Bio-tek, Inc., United States) and was used as a PCR template for the amplification of the ITS1 region and quantitative PCR. Fungal ITS gene primer ITS1F (F) (5′ CTTGGTCATTTAGAGGAAGTAA 3′) and primer ITS2(R) (5′ GCTGCGTTC>TTCATCGATGC 3′) (White et al., 1990; Gardes and Bruns, 1993) were used in the PCR reaction. ITS1 and ITS2 primers yield similar results and are suitable as DNA metabarcoding markers (Blaalid et al., 2013). Barcode sequences (6-bases) were ligated to the 5′ end of the reverse and forward sequencing primers during the process of primer synthesis prior to PCR amplification. All PCR reactions were performed with the following conditions: an initial hot start incubation (5 min at 95°C) followed by 30 cycles of denaturation at 94°C for 30 s, annealing at 55°C for 35 s and extension at 72°C for 30 s, and a final elongation step at 72°C for 8 min. PCR products from three separate amplification reactions were pooled and then purified using TIANquick Midi Purification Kit (Tiangen Biotech Co., Ltd., Beijing, China). Amplicon libraries were then generated using NEB Next R Ultra TM DNA Library Prep Kit for Illumina (NEB, United States) following the manufacturer’s recommendations. PCR reaction, amplicon library preparation, and high-throughput sequencing using the Illumina HiSeq 2500 platform (Illumina Inc., San Diego, CA, United States) following the manufacturer’s instructions were performed by AuGCT Biotechnology Co., Inc., Beijing, China.

### Fungi-Specific Quantitative PCR

Fungi-specific 18S rRNA gene quantitative PCR (qPCR) was performed using the method described by Taylor and Cunliffe (Taylor and Cunliffe, 2016). The primers FR1 5′-AICCATTCAATCGGTAIT-3′ and FF390 5′-CGATAACGAACGAGACCT-3′ (Vainio and Hantula, 2000; Prévost-Bouré et al., 2011) were used. A 10 μl reaction mixture contained 5 μl of 2X SybrGreen qPCR mix (GENE-BETTER^TM^, Beijing, China), 0.25 μl of each primer (10 pmol μl^-1^), 1 μl of DNA template (ca. 52–56 ng/μL) and 3.5 μl nuclease-free molecular-grade water. A CFX Connect^TM^ Real-Time System (Bio-Rad Laboratories, Inc., Hercules, CA, United States) was used to perform qPCR. The following qPCR regime was used: denaturation at 94°C for 3 min, with 40 cycles at 94°C for 10 s, annealing at 50°C for 15 s, elongation and acquisition of fluorescence data at 72°C for 20 s. Standard curve was constructed using known amounts of target template generated by PCR amplification of the target gene from genomic DNA of *Rhodosporidium* sp. TJUWZ4 (CGMCC #2.5689, GenBank No: KT281890.1). The PCR product was purified by TIANquick Midi purification Kit (Tiangen Biotech (Beijing) Co., Ltd.) and its sequence was verified through sequencing (Beijing Genomics Institution, China). The target DNA fragment was then cloned into pTOPO-T Vector by Zero Background pTOPO-TA Cloning Kit following manufacturer’s instructions (GENE-BETTER TM, Beijing). A E.Z.N.A.^TM^ Plasmid Midi Kit (Omega Biotek, Inc., United States) was used to isolate the plasmid vector from the transformed *Escherichia coli* DH5α competent cells (Solarbio^®^ LIFE SCIENCES, Beijing, China), and the concentration of extracted plasmid DNA was determined using the NanoDrop ND-1000 spectrophotometer (Nanodrop Technology, Thermo Fisher Scientific Inc.). The plasmid was linearized with HindIII [Takara Biomedical Technology (Beijing) Co., Ltd.] and the resulting plasmid was gel purified using TIANquick Midi Purification Kit (Tiangen Biotech Co., Ltd., Beijing, China) and its concentration was measured on a Nanodrop ND-1000 spectrophotometer. Appropriate dilutions of purified linear plasmid were done to obtain gene copy numbers ranging from 10^3^ to 10^8^ copies/L which were then stored at -20°C. A 1 μL template from each dilution was used to prepare standard curve for each qPCR assay. The average qPCR efficiency was 90.9%.

### High-Throughput Sequence Analysis

The base call of each sequence read was inspected and filtered for quality control purpose. The raw reads were processed following the pipeline of Mothur (Schloss et al., 2009). All reads matching the barcodes (maximum mismatch = 1) were retained as well as reads with a maximum 3 bases mismatch to the primers. Reads were then trimmed by removing the sequencing adaptor, barcodes, and primer sequences. These reads were further screened by using the following thresholds: (i) minimum average quality score of 25; (ii) minimum read length of 200 bp; (iii) sequences containing no ambiguous bases; and (iv) maximum homopolymers of 8 bp (Liu et al., 2015). Sequence read pairs were merged using FLASH (Magoc and Salzberg, 2011) and compressed based on 97% similarity. Chimeric clusters were removed using *de novo* in USEARCH and unique clusters were subjected to BLAST analysis. The databases for fungal ITS sequences are not complete, so we added few more steps in the annotation of sequences. We used the BLASTn method with NT database (e < 1e-5, coverage > 80%) and the specialist fungal database, the UNITE database^1^ (accessed in 2016) by classify.seqs() in Mothur with a similarity threshold of 0.8, to assign taxonomy to OTUs (Abarenkov et al., 2010). The search results were then mutually compared, and when the two databases results did not agree, the highest query coverage and percentage ID were chosen as a match. The other eukaryote sequences were removed using remove.lineage() in Mothur and those sequence which matched <100% to kingdom Fungi in UNITE database were removed from the dataset using perl script developed in-house. Sequences are available from the European Nucleotide Archive (Bioproject accession code: PRJNA341916).

### Statistical Analysis

Normal distribution of the data was checked using Shapiro–Wilk test of normality. *Post hoc* tests according to Nemenyi-tests for multiple comparisons of (mean) rank sums of qPCR abundance of samples across each month were performed after a Kruskal–Wallis test. To explain the relationship between data (qPCR abundance, order abundance) and gradients (month, section, and depth) we conducted Kruskal-Wallis test. Correlation between OTU data at phyla level and environmental data was determined using Pearson’s correlation test. A heatmap was generated with OTU relative abundance data and samples were clustered based on Bray-Curtis distance to visualize the β-diversity. Monte Carlo permutation test was performed followed by a constrained canonical correspondence analysis to estimate the correlation between OTU relative abundance and selected environmental factors. The above statistical tests were performed using vegan and status packages in R software (R version 3.3.1). Shannon diversity index for each sample was calculated at a 3% dissimilarity level using Mothur (Schloss et al., 2009) as the measure of α diversity. A network was constructed with CoNET (Faust et al., 2012) – a plugin in Cytoscape software following the method described elsewhere (Debroas et al., 2017). We employed an ensemble approach combining four different measures: two measures of dissimilarity (Bray–Curtis (BC) and Kullback–Leibler (KLD)) and two measures of correlation (Pearson and Spearman correlation). The dataset of 54 samples used in network analysis contained OTUs in rows and samples in columns. The rows were divided by their sum prior to computation of BC and KLD measures. The row minimum occurrence parameter was set at 20. To test the statistical significance of the edge scores, we computed measure- and edge-specific permutation and bootstrap score distributions with 1000 iterations each. The p-values were then computed by z-scoring the permuted null and bootstrap confidence interval using pooled variance. Edges with scores not within 95% confidence interval of the bootstrap distribution were removed.

## Results

### Abundant and Dominant Planktonic Fungi in the Coastal Waters

**FIGURE 2:**
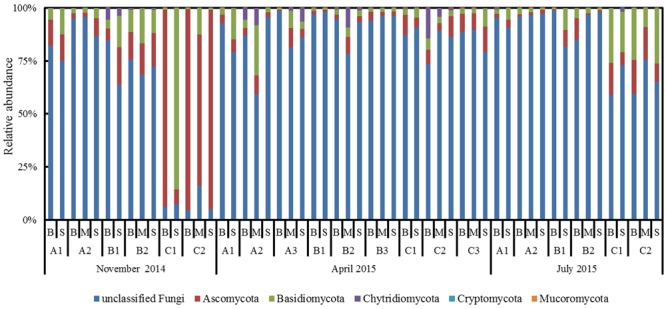
Relative abundances of the planktonic fungal phyla across all samples collected from Qinhuangdao coastal area.

We obtained high quality 6,072,738 reads with a read length of 200–440 base pairs from the Illumina HiSeq 2500 platform sequencing. The number of sequences from each sample ranged from 7,153 to 431,051 with an average number of 112,456 ± 96,784 (mean ± SD). The total sequences obtained after filtering were assigned to 4,362 OTUs. Among them, 1,483 OTUs (1,701,463 sequences) covered five known fungal phyla namely Ascomycota, Basidiomycota, Chytridiomycota, Mucoromycota, and Cryptomycota, 20 classes, 58 orders, 111 families, and 283 genera. Of these five known phyla, Ascomycota was the most abundant and accounted for 20.82% of the total 4,362 OTUs, covering 1,182,597 sequence reads (19.47% of total 6,072,738 reads). Basidiomycota, Chytridiomycota, Mucoromycota, and Cryptomycota represented 9.72, 2.64, 0.64, and 0.18% of the OTUs (4,362), respectively. Of particular interest, the phylum Cryptomycota and order Mortierellales of phylum Mucoromycota were detected first time in the coastal water column through high-throughput sequencing analysis where Mucoromycota was detected in 32 samples and only nine samples featured Cryptomycota. Within the most abundant top 50 OTUs, 12 OTUs (OTU3 OTU4, OTU7, OTU14, OTU16, OTU20, OTU21, OTU26, OTU32, OTU33, OTU38, and OTU40) were assigned to the Ascomycota, three OTUs (OTU6, OTU36, and OTU37) to the Basidiomycota and one OTU (OTU52) to the fungal parasite Chytridiomycota that were recently reported as important parasite of phytoplankton (Gutiérrez et al., 2011; Hassett and Gradinger, 2016; Jephcott et al., 2017). Nevertheless, more than 60% of the fungal sequences (4,371,275) could not be classified to any fungal phyla (**Figure 2**) based on the database information, likely suggesting undescribed fungal sequences in the coastal waters of Bohai Sea.

**FIGURE 3:**
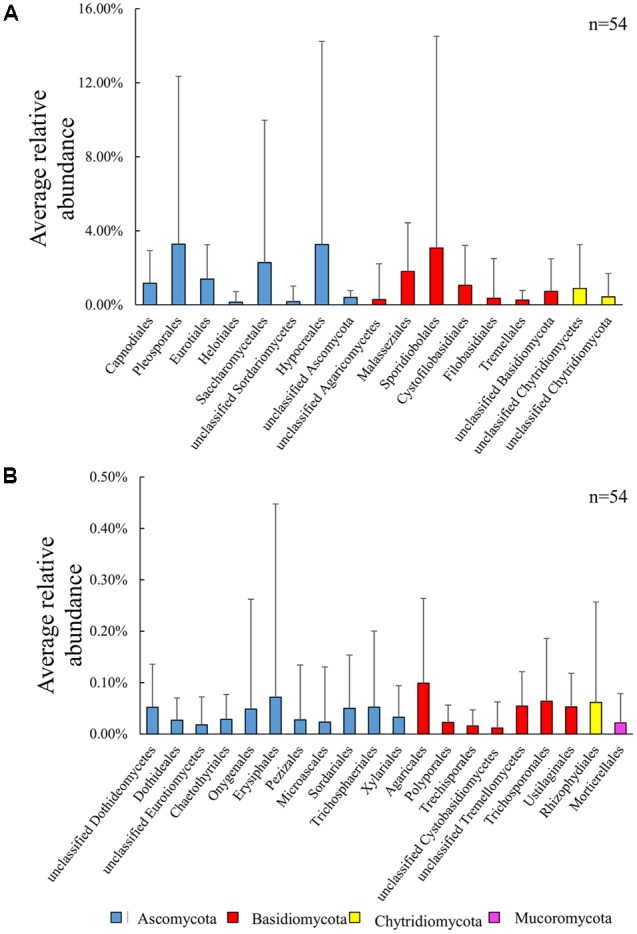
Average relative abundance of planktonic fungal taxa in samples collected from Qinhuangdao coastal area from November 2014 to July 2015. Fungal taxon < 0.01% have been omitted from the figure. Average relative abundance was estimated for each fungal taxon by dividing the total relative abundance across all samples by the number of samples (*n* = 54). The error bars onto each of the columns show the standard deviation (calculation across 54 samples) for each average value. **(A)** Taxa with average relative abundance > 0.1% and **(B)** taxa with average relative abundance ≤ 0.1%.

Within the five phyla, several taxa were present during the entire sampling period (**Figure 3**). Of these, several orders were frequently dominant with proportions in total sequences more than 1% (**Figure 3A**). Among these dominant orders, Hypocreales was found to be the most abundant (139 OTUs, 29.18% of fungal sequences), followed by Pleosporales (119 OTUs, 12.76%), Sporidiobolales (37 OTUs, 8.91%), Saccharomycetales (53 OTUs, 7.81%), Eurotiales (115 OTUs, 7.57%), Malasseziales (38 OTUs, 6.22%), Capnodiales (36 OTUs, 6.03%) and Cystofilobasidiales (11 OTUs, 2.84%). These eight dominant orders accounted for 81.32% of the total classified sequences that were generated within the five known phyla. Each dominant order composed of multiple OTUs was detected in the majority of the samples (Supplementary Figure S1). Notably, we found the order Mortierellales (phylum Mucoromycota) within the group of taxa with average relative abundance less than 0.1% (**Figure 3B**). The percent frequencies of Capnodiales, Pleosporales, Eurotiales, Saccharomycetales, Hypocreales were lowest (≤0.002%) in samples of section A2 in November while of Malasseziales, Sporidiobolales, and Cystofilobasidiales, the lowest were in the surface sample of section C2 in November, and mid-depth samples of section A2 in July, respectively. The peak percent frequencies (≥0.5%) of these orders were observed during November (C1B, C2B, C1B, C2M, C1B, C1S, and B1B, respectively), except Malasseziales which showed peak during July (B1S).

### Abundance of Planktonic Fungi

**FIGURE 4:**
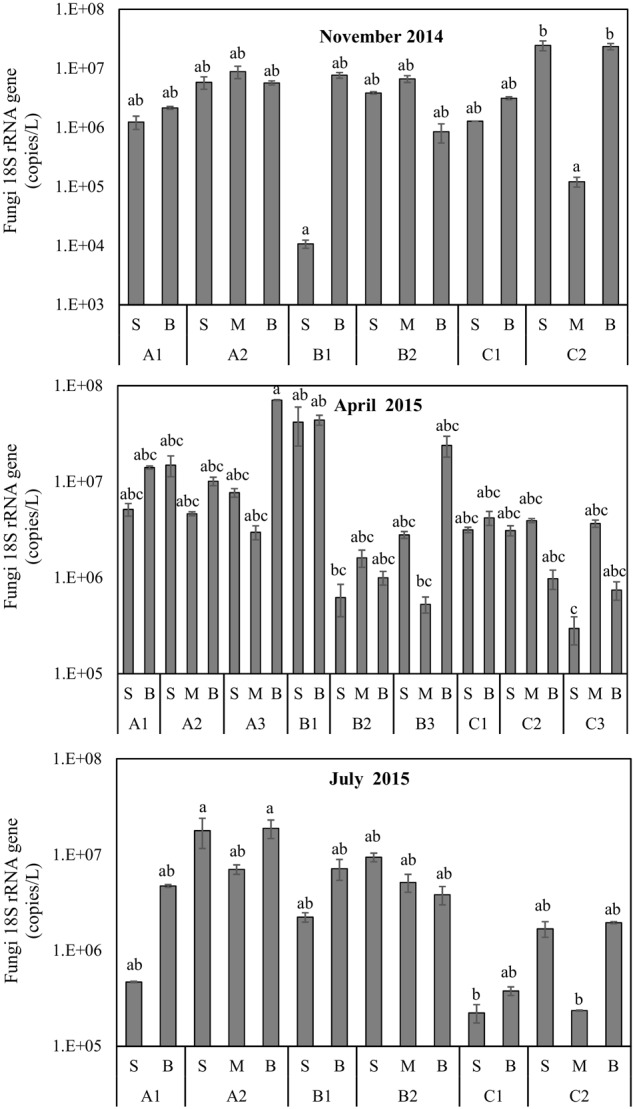
Planktonic fungal abundance determined by Fungi-specific 18S rRNA gene qPCR of plankton DNA samples collected from Qinhuangdao coastal area from November 2014 to July 2015. Samples of each month with significant mean differences (*P* < 0.05) based on Kruskal–Wallis *post hoc* test (Nemenyi-tests) are marked in different letters (a, b, c). Thus, samples (bars) that are not significantly different are indicated with same letters (for e.g., ‘abc’) and sample pairs with significant differences will have different letters.

The results of planktonic fungal abundance estimated using qPCR with the Fungi-specific 18S rRNA gene-specific primers are shown in **Figure 4**. The mean abundance of samples across the seasons in Qinhuangdao coastal areas was 8.20 × 10^6^ copies/L. Of all samples, the lowest and the highest abundances were in November (B1S, 1.07 × 10^4^ copies/L) and in April (A3B, 7.09 × 10^7^ copies/L), respectively. Overall, the abundance varied significantly across sections (*P* < 0.05, Kruskal–Wallis test). Notably, the mean abundance in section A was higher than section C (*P* < 0.05, Nemenyi-tests) in July but not in November and April (*P* > 0.05, Nemenyi-tests). The results suggest the differential influence of riverine inputs and anthropogenic activities especially during the warm and wet season (July).

### Spatiotemporal Dynamics and Drivers of Planktonic Fungi in Coastal Waters

**FIGURE 5:**
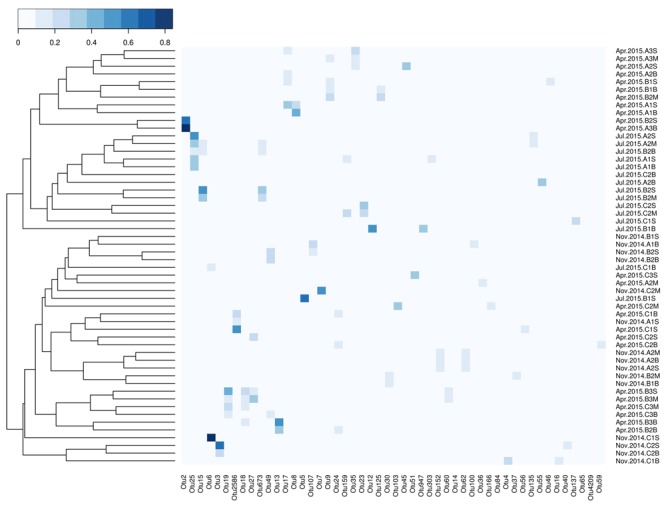
Heatmap of the top 50 OTUs based on their relative abundance across samples from Qinhuangdao coastal area. Sample relationships are shown through clustering based on Bray–Curtis dissimilarity measure. Of these OTUs, the classified OTUs belonged to phyla Ascomycota (OTU# 3, 4, 7, 14, 16, 40, 73), Basidiomycota (OTU# 6, 36, 37, 65), and Chytridiomycota (OTU# 59). The color-key indicate the range of relative abundance of the OTUs.

Cluster analysis based on the relative abundance of top 50 OTUs (accounted for 66.04% of total sequences) revealed OTU composition dissimilarities across the samples in Qinhuangdao coastal area (**Figure 5**). Notably, the OTU composition within samples of July was distinctly different from other seasons. Most samples of section A and section B in April had a similar OTU composition to that of samples in July. All the samples of section C in April and November formed a separate cluster. Based on permutation tests of CCA results, the composition of the top 50 OTUs was significantly correlated with co-occurring concentrations of DIP (*P* < 0.001), silicate (*P* < 0.001), and DIN (*P* < 0.005) (**Table 2**).

**Table 2:** Correlations between selected environmental variables and planktonic fungi composition (OTU level) assessed by Monte Carlo permutation test (999 permutations) for canonical correspondence analysis.

Variables	CCA1	CCA2	*r*^2^	*P*-value
Chlorophyll-a	–0.7756	0.6312	0.1055	0.085
DIP	0.1042	0.9945	0.8001	0.001 ^∗∗∗^
silicate	–0.5200	0.8541	0.6989	0.001 ^∗∗∗^
DIN	0.3508	0.9364	0.5888	0.001 ^∗∗∗^

*^∗^*P* < 0.05, ^∗∗^*P* < 0.01, ^∗∗∗^*P* < 0.001.*

**FIGURE 6:**
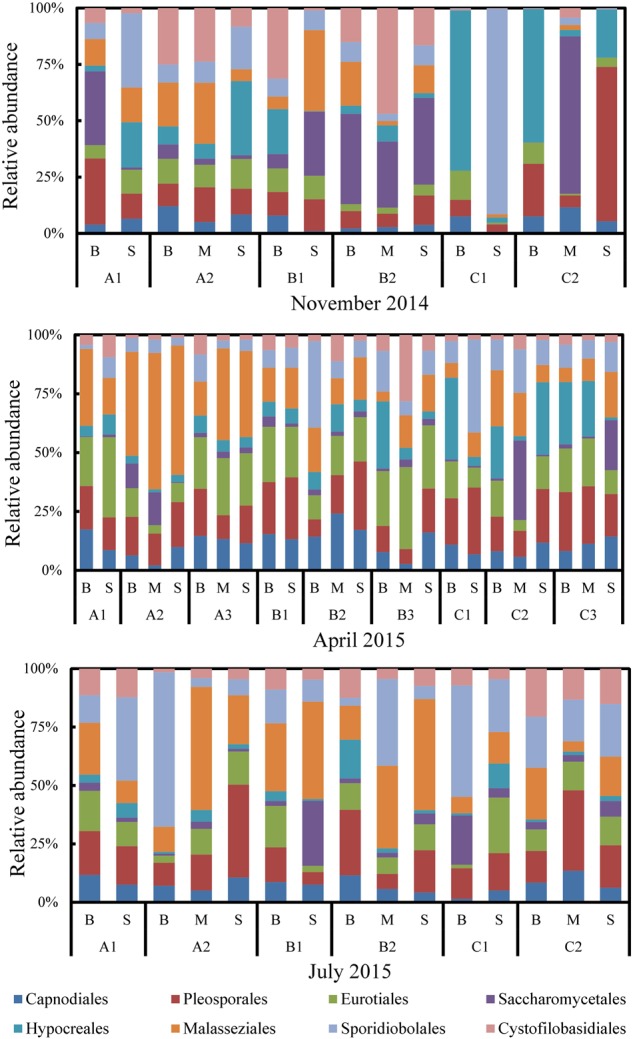
Composition of dominant planktonic fungal orders across all samples collected from Qinhuangdao coastal area.

The proportion of the unclassified fungi was the maximum in all the sections except for section C in November. Of the classified phyla, Ascomycota and Basidiomycota were dominant in all the three sections and seasons (**Figure 2**). Occasionally, Chytridiomycota exhibited higher proportion than Ascomycota and Basidiomycota in April (A3S, B2S, and C2B) (**Figure 2**). Focusing on the relative abundance of eight major orders revealed their dominance across time and space (lateral and vertical) (**Figure 6**). Of the eight orders, Hypocreales varied significantly (*P* < 0.01, Kruskal–Wallis test) across months while Capnodiales, Pleosporales, Eurotiales, and Sporidiobolales varied significantly (*P* < 0.05, Kruskal–Wallis test) across sections.

The changes in Ascomycota and Basidiomycota were closely linked to the changes in DP, PP, DIN, and silicate (**Table 3**). The OTU abundance of Ascomycota and Basidiomycota was greatest when the concentration of these nutrients increased. Additionally, Ascomycota abundance increased significantly (*P* < 0.001, Pearson’s correlation test) when pH of seawater decreased. In contrast, Cryptomycota OTU abundance was greatest when the levels of Chlorophyll *a* increased (*P* < 0.01, Pearson’s correlation test). Mucoromycota OTUs abundance increased when the salinity of seawater increased.

**Table 3:** Correlations between environmental variables and normalized abundance of dominant planktonic fungi phyla.

Phyla	Significant correlations with environmental variable (*P* < 0.05)^∗^
Ascomycota	pH (*P* = 0.001, ρ = -0.485)
	DIP (*P* = 0.021, ρ = 0.314)
	TP (*P* = 0.001, ρ = 0.439)
	DP (*P* = 0.045, ρ = 0.274)
	PP (*P* = 0.000, ρ = 0.508)
	Silicate (*P* = 0.006, ρ = 0.368)
	Nitrite (*P* = 0.000, ρ = 0.607)
	Nitrate (*P* = 0.000, ρ = 0.467)
	DIN (*P* = 0.001, ρ = 0.426)
	DN (*P* = 0.000, ρ = 0.495)
	DON (*P* = 0.016, ρ = 0.328)
Basidiomycota	DIP (*P* = 0.000, ρ = 0.548)
	TP (*P* = 0.000, ρ = 0.502)
	DP (*P* = 0.000, ρ = 0.565)
	PP (*P* = 0.001, ρ = 0.426)
	Silicate (*P* = 0.003, ρ = 0.395)
	Nitrate (*P* = 0.029, ρ = 0.297)
	DIN (*P* = 0.043, ρ = 0.276)
	DN (*P* = 0.048, ρ = 0.271)
Cryptomycota	Chlorophyll *a* (*P* = 0.000, ρ = 0.501)
Mucoromycota	Salinity (*P* = 0.000, ρ = 0.513)

*^∗^Based on Pearson’s correlation analysis.*

The α-diversity results of plankton fungi in Qinhuangdao coastal area are presented in **Table 4**. The Shannon index varied considerably across seasons and sections. Across all seasons and sections, the highest variance (σ^2^ = 0.99) in the planktonic fungi Shannon index was noted in section A during April, while the lowest variance (σ^2^ = 0.04) was in section B during November. The maximum Shannon index of 4.4 was observed in section A during April and November (Supplementary Table S2). No significant correlation of Shannon diversity with any of the environmental parameters was obtained (data not shown).

**Table 4:** Shannon diversity index for the planktonic fungi in the coastal waters of Qinhuangdao.

	November 2014			April 2015			July 2015		
Section	A	B	C	A	B	C	A	B	C
Mean	3.91	3.80	2.28	3.27	2.77	3.41	2.97	2.35	3.39
Min.	3.56	3.60	1.19	1.03	2.09	2.55	2.65	1.56	2.88
Max.	4.42	4.20	3.00	4.41	3.54	3.99	3.29	3.63	4.11
Variance (σ^2^)	0.11	0.04	0.44	0.99	0.31	0.16	0.07	0.57	0.18

Overall, the results suggested the prominence of planktonic fungal phyla, Ascomycota and Basidiomycota, in the coastal waters of Qinhuangdao, with their abundance greatly influenced by some co-occurring nutrient levels. Distinct changes in the relative abundances of major orders across seasons and transects were evident.

### Planktonic Fungi Assemblages

**FIGURE 7:**
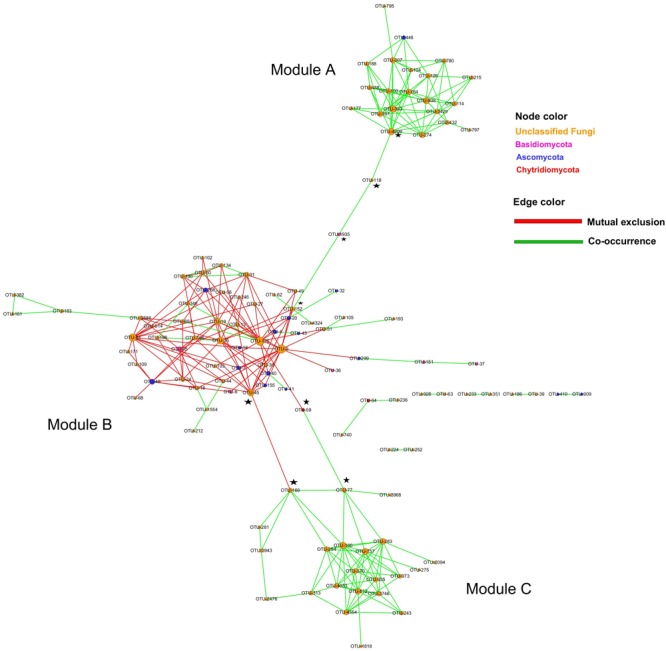
Planktonic fungi network showing significant co-occurrence and mutual exclusion relationships among abundance of clades in the Qinhuangdao coastal waters. Circles indicate OTUs; circle width is a function of the degree of node. The keystone OTUs are indicated by a five pointed star in black.

To identify the possible assemblages existing among planktonic fungal OTUs in the coastal water system, a network was constructed and inferred. The resulting network (**Figure 7**) was composed of 117 nodes with clustering coefficient of 0.185 and 4.6 average number of neighbors. Analysis and visualization of the network revealed a planktonic fungal community consisting of three modules (A, B, and C) and several hubs (**Figure 7**). Two modules (A and C) were composed of co-occurring OTUs, while one module (B) relatively a larger sub-network than the other two clustered OTUs showed mostly mutual exclusion. Interestingly, the network also revealed several keystone species that were assigned to phyla Ascomycota (OTU152, OTU118, OTU4209, OTU77, OTU45, and OTU166), Basidiomycota (OTU1935) and Chytridiomycota (OTU59) at ≤0.8 identity threshold. Thus, species belonging to these phyla probably have roles in key metabolic steps within the fungal community and their further characterization would throw much information on their physiological and metabolic potential.

## Discussion

### Diversity and Abundance of Planktonic Fungi

The present work explicates a high planktonic fungal diversity featured by the phyla Ascomycota and Basidiomycota and to a lesser extent the phyla Chytridiomycota, Cryptomycota, and Mucoromycota in the coastal waters. The dominance of Ascomycota, Basidiomycota, and Chytridiomycota phyla has been previously described in the coastal marine habitats (Gao et al., 2010; Taylor and Cunliffe, 2016; Picard, 2017). The overall diversity in fungal OTU composition in the Qinhuangdao coast of Bohai Sea was relatively higher compared to other studies, based on DNA sequencing, from different marine ecosystems (Richards et al., 2012; Manohar and Raghukumar, 2013), including coastal waters (Gao et al., 2010; Taylor and Cunliffe, 2016), surface open ocean (Wang et al., 2014), deep open ocean (Bass et al., 2007), and deep ocean sediments (Bass et al., 2007; Orsi et al., 2013; Orsi et al., 2017). Two fungal clades: Mortierellales (phylum Mucoromycota) and basal zoosporic fungi Cryptomycota are reported for the first time in the coastal waters. The members of Mortierellales are the most common Zygomycete fungi encountered in soil (Benny et al., 2016) and their presence in coastal waters perhaps implies their terrestrial-marine transition by terrestrial runoff or tidal action. Terrestrial forms of fungi have been found to be active in marine environment owing to physiological versatility (Raghukumar and Raghukumar, 1999). Our study, which revealed a high fungal diversity and OTU abundance, provides the basis of future studies on planktonic fungi that may challenge the historical view of fungi constituting a small fraction of total eukaryotes in coastal waters (Monchy et al., 2012).

The spatiotemporal changes in the planktonic fungi abundance estimated from Fungi-specific 18S rRNA gene based qPCR analysis suggested that their assemblages in the water column of the Qinhuangdao Coast of Bohai Sea are active and fluid. A similar feature of planktonic fungi abundance from the multi-year assessment was noted in the English Channel (Taylor and Cunliffe, 2016). The abundance of planktonic fungi, mostly higher in the coastal waters than in pelagic waters, is often attributed to the high carbon from autochthonous primary production and allochthonous (terrestrially derived) production in coastal region (Newell, 1982; Gao et al., 2010; Gutiérrez et al., 2011; Wang et al., 2014). In the nearshore water column of Qinhuangdao coastal area, the abundance was higher at the bottom than on the surface. Similarly, in the upwelling region of Chile, planktonic fungi biomass increased with depth in the top 15 m (Gutiérrez et al., 2011). Several lines of evidence suggested that fungi are relatively abundant in marine sediments (Orsi et al., 2013; Xu et al., 2014; Taylor and Cunliffe, 2015; Hassett and Gradinger, 2016; Li W. et al., 2016) than in the water column (Taylor and Cunliffe, 2016). An earlier study (Richards et al., 2012) provided the analogous explanation for the higher abundance at the nutrient rich bottom of the water column includes direct impact of the tidal action, releasing fungi from sediments into the water column, thus enabling these osmotrophs to populate therein.

The biomass (abundance) of fungi is an important indicator of its ecological role in marine habitats (Damare and Raghukumar, 2008). Previous studies have shown that fungal biomass in the coastal waters represent a considerable portion of microbial biomass and often of the similar order of magnitude to that of marine prokaryotes. For example, the planktonic fungi biomass was found to be comparable to prokaryotes biomass in the upwelling ecosystem off central-southern Chile (Gutiérrez et al., 2011). Likewise, in the West Pacific Warm Pool, Basidiomycota and bacterioplankton were in the similar order of the magnitude of DNA quantity (Wang et al., 2014). Similarly, the Fungi-specific 18S rRNA gene qPCR abundance (on average 8.20 × 10^6^ copies/L) and bacterial cell numbers (on average 2 × 10^6^ cells/L, data not shown) in the coastal waters of Qinhuangdao showed a roughly similar order. However, it should be noted that fungal cell numbers cannot be estimated from the Fungi-specific 18S rRNA gene qPCR abundance (Taylor and Cunliffe, 2016), and thus our comparison of fungal and bacterial abundances in the Qinhuangdao coastal waters is just an approximation and does not reflect an absolute interpretation. Moreover, Fungi-specific 18S rRNA gene qPCR abundance in our study only served as a proxy for planktonic fungi biomass because it is not possible to estimate the carbon biomass from qPCR abundance (Taylor and Cunliffe, 2016). Nevertheless, the planktonic fungi abundance changes, both over space and time, in the present study were quite pronounced (**Figure 4**) which likely indicated fungal carbon turnover in the Qinhuangdao coastal waters. Thus, we speculate that the dynamic planktonic fungi biomass is one of the significant components of the coastal carbon cycle, and like prokaryotes, fungi too have an important contribution to the coastal secondary production.

### Forcing Factors and Prediction of Ecological Roles of Planktonic Fungi

As one of the most variable marine ecosystems, coastal waters are usually characterized by a high plankton diversity and high primary production (Jickells, 1998; Danovaro and Pusceddu, 2007). Of particular notice, some pollutants that are washed off from upstream may lead to nutrient enrichments in both sediments and water column and may have detrimental effects on species abundance and fungal community composition. Die-off from dominant species resulting from eutrophication would stimulate the appearance of opportunistic species and may contribute to the increase of total diversity (Johnston and Roberts, 2009). In this study, the fungal OTU composition and the dominant phyla were closely linked with the co-occurring multiple nutrients (**Tables 2, 3**); thus, we hypothesize that dominant species death probability rise when the nutrient concentration reduces, to allow the growth of the opportunistic species, eventually resulting in an increase in diversity.

The diversity and OTU abundance of planktonic fungi were largely regulated by the variations in the availability of several of potential growth substrates, for example, the changes in organic and inorganic nitrogen-rich substrates impacted the abundance and diversity at Station L4 of Western English Channel (Taylor and Cunliffe, 2016). In particular, Ascomycota diversity increased with the ammonia and phosphate concentrations, unlike Basidiomycota which did not link to changes in any of the co-occurring environmental parameters at Station L4 (Taylor and Cunliffe, 2016). However, in our study, both Ascomycota and Basidiomycota were impacted by the inorganic nitrogen and phosphorous sources (**Table 3**) which are key nutrients for phytoplankton growth. This suggests that these two phyla seem to be chiefly involved in processing phytoplankton detritus when substrate availability is high (phytoplankton bloom) in the water column of Qinhuangdao coastal area. Several studies have shown the direct involvement of fungi in nitrogen metabolism, including ammonia assimilation and nitrite ammonification (Wegley et al., 2007). Besides processing the phytoplankton detritus, planktonic fungi may also play a role in the water column nitrogen metabolism because of their significant relationship with nitrogen in this study (**Tables 2, 3**). Thus, the ecological function of planktonic fungi should not be ignored in the process of the nitrogen cycle.

The sampling sites in our study were influenced by freshwater discharge from the Tang River, Yang River, and Dapu River, which may supply terrigenous organic matter to the coastal zone. These rivers together with the tides, rain, and the wind mostly affect the coastal ecosystem. With different mean diversity of planktonic fungi in each of the three different sections (**Table 4**) and significantly different qPCR abundance across sections, we speculate that Qinhuangdao coastal ecosystem is remarkably affected by different rivers and their inputs. Considering that fungi have been found to be responsible for degrading mainly terrigenous detritus in marine/terrestrial ecotones (Hyde et al., 1998; Pointing and Hyde, 2000; Kis-Papo, 2005), the relatively higher fungal diversity in the nearshore is most likely associated with the availability of terrestrial remnants from rivers. A similar trend of higher molecular richness nearshore than offshore was reported for sea waters of the Hawaiian coast and coastal upwelling ecosystem of central Chile (Gao et al., 2010; Gutiérrez et al., 2010; Wang et al., 2014).

The members of Chytridiomycota are zoosporic fungi that are either saprotrophs or parasites and are one of the important primary consumers in aquatic food-web (Frenken et al., 2017; Jephcott et al., 2017). Similarly, the members of phylum Cryptomycota (historically named as Rozellomycota), considered as the most basal clade of fungi, are also characterized as unicellular endoparasites of algae (Corsaro et al., 2014), and some reviews have suggested their role as hyperparasites of parasitic fungi Chytridiomycota (Gleason et al., 2012, 2014). Yet, both these groups of marine fungal parasites are poorly understood (Gleason et al., 2011), including their interactions with phytoplankton (Scholz et al., 2016). Although a tripartite interaction between the members of Chytridiomycota, Cryptomycota, and phytoplankton has been proposed (Gleason et al., 2014), such model has not been established in marine ecosystems so far. Based on this model, the presence of the members of Cryptomycota in the coastal waters would result in a reduction of the number of Chytridiomycota members eventually favoring phytoplankton bloom. We found a positive association of Cryptomycota OTU abundance and Chlorophyll *a* (**Table 3**) in our study but did not observe any relationship between Chytridiomycota and Chlorophyll *a*. Previous findings show that the cells of Cryptomycota are frequently found on diatoms and they acquire cell wall materials from their host (Jones et al., 2011). Thus, we hypothesize that some members of Chytridiomycota are playing a saprotrophic role, and members of Cryptomycota were either hyperparasites of the parasitic members Chytridiomycota or algal parasites. Previous studies have shown the frequent occurrence of the brown tide of picophytoplankton algae in Qinhuangdao coastal sea (Zhang et al., 2012; Yang et al., 2016; Qiao et al., 2017). We confirmed such brown tide outbreak in 2015 by comparing the color difference between brown tide water and non-brown tide water in the true color composite image of HJ-1 (data not shown). Moreover, the appearance of abnormal ocean color has been reported when Chlorophyll *a* was ≥2.5 mg/m^3^ (Yang et al., 2016). In our study, the content of Chlorophyll *a* in the seawater reached 5.21 μg/L in July. These findings indirectly confirm the occurrence of phytoplankton bloom in the Qinhuangdao coastal water in 2015. We also noted high silicate content in seawater in July 2015, with concentration reaching up to 311.47 μg/L (Supplementary Table S1), perhaps resulting from the degradation of algal bloom detritus by saprotrophic members of Chytridiomycota leading to the release of silicates into the seawater. The positive association between Cryptomycota and Chlorophyll *a*, the occurrence of algal bloom, and release of silicate during an algal bloom, taken together suggest the regulation of the parasitic members of Chytridiomycota by the hyperparasitic members of Cryptomycota, ultimately favoring the algal bloom. Our findings provide a plausible evidence of the existence of a tripartite interaction in the Qinhuangdao coastal waters. Thus, we propose that the members of Chytridiomycota and Cryptomycota might play a significant role in the nutrient and energy flow in the water column food web through a tripartite interaction with phytoplankton.

### Network Topology and Fungal Assemblages

Co-occurrence networks are essential for the understanding and management of the dynamics of the individual group members and of the entire ecosystem *per se* (Gaedke, 2007; Faust and Raes, 2012). Among the many topological indices, degree, closeness and redundancy in the networks provide information on the robustness of the community and its likely ability to resist change. Moreover, the highly connected phylotypes sometimes called hubs or keystones, characterized by their high degree, high closeness, and low betweenness, are predicted to perform key metabolic steps within the community (Mondav et al., 2017). In our planktonic fungi network at Qinhuangdao coastal waters, several hubs with high degree and high closeness but high betweenness were identified. Therefore, these hubs with their high betweenness and taxonomically close to adjacent OTUs exhibit qualities associated with redundancy or ‘niche overlap.’ The elimination of such hubs will have little or no effect on the community function and are unlikely the keystone species. Thus, keystone species are ecologically those that would cause (disproportionate) disruption to a network if lost, even though they are sometimes described statistically as hubs (Berry and Widder, 2014). Interestingly, we found several OTUs belonging to phyla Ascomycota, Basidiomycota, and Chytridiomycota that likely fit the description of keystone species in our network (putative keystones indicated in **Figure 7**). Although, these keystone fungal-OTUs could not be assigned to the genus and species levels because of limited fungal database information, we speculate that species within these phyla play crucial role in the fungal assemblages. The loss of these fungal OTUs would fragment the network and/or were the only phylotypes that have a critical metabolic function. Thus, the loss of any of the identified keystone fungal OTUs from the coastal water of Qinhuangdao could affect significant changes in nutrient cycling.

Statistically, none of the predicted keystone fungal OTUs in our network had a high degree, this means the high degree is a poor predictor of ‘keystoneness’ in coastal waters similar to soil microbial ecosystem (Mondav et al., 2017). Our networks also revealed both negative and positive interactions among the OTUs; and of the three modules, module B was relatively more complex and taxonomically diverse than modules A and B which displayed only positive interactions. Our co-occurrence fungal network reflected niche processes that drive coexistence and diversity maintenance within fungal communities in coastal waters, and showed co-occurring species pairs and assemblages that shared similar ecological characteristics, besides keystone species. The three modules and keystone species mostly consisted of unclassified Fungi (>0.8 sequence similarity threshold), which clearly suggest that further characterization and identification of these novel fungal OTUs are critical for providing a better knowledge of the fungal taxonomic groups and their cryptic role in coastal waters. Clearly, further studies are needed to identify the hubs and keystone fungal species and characterize their metabolic profiles to obtain a better insight into the coastal fungal community network. As per the knowledge of the authors, this is the first report of planktonic fungi network, which will form the basis of future studies on inferring ecological characteristics of poorly understood or non-culturable fungal taxa and their role in nutrient cycling in the marine ecosystems.

To conclude, the spatiotemporal analysis of Qinhuangdao coastal waters of the Bohai Sea has shown that planktonic fungi have a high diversity and abundance and thus they are speculated to play a significant role in the carbon and nitrogen cycling in the water column. The molecular diversity of dominant phyla exhibited a positive relationship with dissolved nitrogen, dissolved and particulate phosphorus, and silicate. We also proposed that riverine inputs have a potential influence on the diversity and abundance of the planktonic fungi, and reported the presence of Mortierellales, the common Mucoromycetes fungi, positively linked with salinity, in the coastal water column. This study provides the hypothesis of a tripartite interaction within members of Cryptomycota, Chytridiomycota, and phytoplankton, and suggests further studies for its validation. Furthermore, the co-occurrence analysis to the planktonic fungal system provided valuable information for characterizing the functional distribution, ecological interactions of fungi at the community scale, and identified ecological traits of poorly defined phylotypes that co-occur with well-characterized fungal taxa. Altogether, our findings clearly demonstrated the role of planktonic fungi in nutrient cycling as saprotrophs and parasites manifested by their high abundance, diversity, and co-occurrence in the water column and emphasize their ecological significance in the realm of the coastal ocean environment.

## Author Contributions

YW conceived the study, performed the molecular work, and statistical analysis. BS conceived the study, analyzed the data, performed the statistical analysis, and wrote the paper. YH and NX collected the field data and samples. GW conceived the study and revised the paper. All authors have approved the submission of the article.

## Conflict of Interest Statement

The authors declare that the research was conducted in the absence of any commercial or financial relationships that could be construed as a potential conflict of interest.

## Footnotes

1 https://unite.ut.ee/repository.php
