# Supplementary material for: Spatiotemporal Distribution and Assemblages of Planktonic Fungi in the Coastal Waters of the Bohai Sea
Source: Front Microbiol. 2018 Mar 28;9:584. doi: 10.3389/fmicb.2018.00584 (PMC5882831; doi:10.3389/fmicb.2018.00584)
Supplement: Supplementary file 1 [file Table_1.docx]

Supplementary Material

Spatiotemporal Distribution and Assemblages of Planktonic Fungi in the Coastal Waters of the Bohai Sea

**Yaqiong Wang^1, 2, †^, Biswarup Sen^1, †^, Yaodong He^1^, Ningdong Xie^1, 3^, Guangyi Wang^1, 4, *^**

^1^ Center for Marine Environmental Ecology, School of Environment Science and Engineering, Tianjin University, Tianjin, China

^2^ School of Ecology, Environment and Resources, Qinghai University for Nationalities, Xining, China

^3^ Duke Marine Laboratory, Nicholas School of the Environment, Duke University, Durham, NC, United States

^4^ Key Laboratory of Systems Bioengineering (Ministry of Education), Tianjin University, Tianjin, China

*** Correspondence:** Corresponding Author: [gywang@tju.edu.cn](mailto:gywang@tju.edu.cn)

^†^These authors contributed equally to this work

**Supplementary Table S1 The concentrations of nutrients in the seawater samples collected from the Qinhuangdao Coast off Bohai Sea.**

| Samples | Chl *a* | DIP | TP | DP | PP | DOP | Silicate | Nitrite | Nitrate | NH_4_^+^ | DIN | DON | DN |
| --- | --- | --- | --- | --- | --- | --- | --- | --- | --- | --- | --- | --- | --- |
| S11A1B | 2.59 | 24.53 | 58.89 | 42.81 | 16.08 | 18.28 | 647.82 | 67.62 | 337.71 | 1.36 | 406.68 | 148.92 | 555.61 |
| S11A1S | 4.85 | 22.92 | 70.87 | 36.95 | 33.92 | 14.03 | 552.82 | 23.70 | 393.80 | 0.13 | 417.63 | 103.70 | 521.33 |
| S11A2B | 2.03 | 7.60 | 34.75 | 24.31 | 10.45 | 16.71 | 416.82 | 10.71 | 255.54 | 0.82 | 267.06 | 62.88 | 329.94 |
| S11A2M | 2.12 | 2.52 | 29.23 | 18.79 | 10.45 | 16.27 | 468.99 | 16.03 | 205.61 | <D.L | 221.64 | 85.95 | 307.59 |
| S11A2S | 1.90 | 8.40 | 17.86 | 15.80 | 2.06 | 7.40 | 469.24 | 2.42 | 242.36 | 0.38 | 245.16 | 102.58 | 347.74 |
| S11B1B | 3.22 | 22.68 | 118.03 | 31.55 | 86.48 | 8.87 | 723.02 | 51.33 | 302.67 | 81.61 | 435.61 | 117.79 | 553.40 |
| S11B1S | 2.58 | 24.61 | 54.75 | 32.70 | 22.06 | 8.08 | 723.99 | 49.80 | 298.51 | 84.87 | 433.18 | 344.37 | 777.55 |
| S11B2B | 5.74 | 5.26 | 30.79 | 17.98 | 12.80 | 12.73 | 402.39 | 24.13 | 194.76 | 4.75 | 223.64 | 177.82 | 401.46 |
| S11B2M | 6.10 | 5.02 | 30.96 | 15.11 | 15.85 | 10.09 | 409.45 | 21.01 | 199.76 | <D.L | 220.77 | 112.98 | 333.75 |
| S11B2S | 6.13 | 5.82 | 28.20 | 14.65 | 13.55 | 8.83 | 398.87 | 24.08 | 202.20 | <D.L | 226.28 | 111.35 | 337.63 |
| S11C1B | 1.23 | 83.89 | 263.20 | 97.52 | 165.68 | 13.64 | 756.85 | 49.21 | 515.98 | 7.80 | 572.99 | 744.75 | 1317.74 |
| S11C1S | 2.53 | 86.55 | 237.42 | 111.43 | 125.99 | 24.88 | 845.75 | 42.60 | 427.68 | 6.79 | 477.07 | 419.75 | 896.82 |
| S11C2B | 0.71 | 5.58 | 36.13 | 16.26 | 19.87 | 10.68 | 422.08 | 48.06 | 193.83 | 0.12 | 242.01 | 643.84 | 885.86 |
| S11C2M | 0.29 | 8.40 | 77.68 | 21.66 | 56.02 | 13.26 | 462.11 | 41.32 | 198.30 | <D.L | 239.62 | 291.51 | 531.14 |
| S11C2S | 0.77 | 5.82 | 72.68 | 16.60 | 56.08 | 10.78 | 378.03 | 38.46 | 214.01 | <D.L | 252.47 | 62.80 | 315.27 |
| S4A1B | 0.91 | 12.78 | 45.26 | 47.62 | 0.00 | 34.84 | 103.35 | 7.50 | 122.72 | 181.41 | 311.64 | 180.31 | 491.95 |
| S4A1S | 0.89 | 3.75 | 46.87 | 36.66 | 10.22 | 32.90 | 146.82 | 8.24 | 128.78 | 181.95 | 318.97 | 466.04 | 785.00 |
| S4A2B | 0.98 | 0.20 | 18.91 | 10.42 | 8.49 | 10.22 | 20.63 | 0.01 | 21.94 | 20.89 | 42.84 | 575.55 | 618.39 |
| S4A2M | 0.90 | 0.10 | 17.73 | 11.39 | 6.34 | 11.29 | 18.62 | 0.12 | 23.17 | 22.23 | 45.52 | 459.67 | 505.19 |
| S4A2S | 0.59 | 1.60 | 17.52 | 9.88 | 7.63 | 8.28 | 13.86 | <D.L | 21.34 | 19.10 | 40.33 | 325.96 | 366.29 |
| S4A3B | 0.81 | 0.53 | 17.84 | 11.60 | 6.24 | 11.08 | 3.36 | 0.26 | 30.47 | 26.68 | 57.41 | 226.79 | 284.20 |
| S4A3M | 0.34 | 2.79 | 16.23 | 13.86 | 2.37 | 11.08 | 7.77 | 0.45 | 26.95 | 24.52 | 51.92 | 74.34 | 126.26 |
| S4A3S | 0.50 | 0.31 | 16.12 | 9.99 | 6.13 | 9.68 | 3.71 | 0.34 | 21.53 | 22.68 | 44.54 | 686.94 | 731.48 |
| S4B1B | 3.31 | 2.89 | 49.77 | 21.71 | 28.06 | 18.82 | 86.92 | 3.57 | 92.52 | 75.41 | 171.50 | 154.49 | 325.99 |
| S4B1S | 4.08 | 5.15 | 39.77 | 22.14 | 17.63 | 16.99 | 107.11 | 3.60 | 96.51 | 65.70 | 165.81 | 215.65 | 381.46 |
| S4B2B | 1.70 | 2.14 | 20.74 | 13.86 | 6.88 | 11.72 | 22.76 | 0.92 | 21.69 | 20.20 | 42.81 | 123.29 | 166.10 |
| S4B2M | 0.80 | 4.29 | 21.49 | 15.47 | 6.02 | 11.18 | 26.15 | 1.35 | 20.42 | 25.78 | 47.55 | 84.84 | 132.39 |
| S4B2S | 1.33 | 19.88 | 10.96 | 19.02 | <D.L | <D.L | 16.74 | 1.07 | 19.51 | 22.81 | 43.39 | 179.81 | 223.20 |
| S4B3B | 1.06 | 5.58 | 15.26 | 13.22 | 2.04 | 7.63 | 0.58 | 0.58 | 31.28 | 19.46 | 51.32 | 155.26 | 206.57 |
| S4B3M | 0.79 | 1.93 | 14.51 | 11.93 | 2.58 | 10.00 | 0.88 | 0.77 | 40.20 | 24.97 | 65.93 | 121.90 | 187.83 |
| S4B3S | 0.46 | 2.03 | 14.18 | 11.71 | 2.47 | 9.68 | 2.66 | 0.89 | 29.46 | 17.61 | 47.96 | 233.82 | 281.79 |
| S4C1B | 9.62 | 12.14 | 57.95 | 25.04 | 32.90 | 12.90 | 43.48 | 2.36 | 40.26 | 10.05 | 52.67 | 219.42 | 272.09 |
| S4C1S | 8.90 | 25.69 | 68.05 | 35.26 | 32.80 | 9.57 | 59.74 | 3.61 | 65.12 | 31.85 | 100.59 | 102.66 | 203.25 |
| S4C2B | 1.88 | 3.54 | 17.62 | 12.03 | 5.59 | 8.49 | <D.L | 0.10 | 10.16 | 15.15 | 25.41 | 106.42 | 131.83 |
| S4C2M | 1.85 | 6.33 | 14.61 | 10.53 | 4.09 | 4.19 | <D.L | <D.L | 15.60 | 13.04 | 28.40 | 126.32 | 154.71 |
| S4C2S | 2.27 | 10.20 | 15.58 | 12.46 | 3.12 | 2.26 | 4.44 | <D.L | 12.59 | 11.65 | 24.08 | 101.69 | 125.77 |
| S4C3B | 1.12 | 0.96 | 14.40 | 14.40 | 0.00 | 13.44 | 1.19 | 0.41 | 28.77 | 28.54 | 57.72 | 145.93 | 203.64 |
| S4C3M | 0.99 | 2.14 | 15.37 | 11.71 | 3.66 | 9.57 | 6.90 | <D.L | 27.57 | 28.58 | 56.03 | 178.97 | 235.00 |
| S4C3S | 0.59 | 0.31 | 12.79 | 11.07 | 1.72 | 10.75 | 5.29 | 0.79 | 31.87 | 42.83 | 75.49 | 152.63 | 228.12 |
| S7A1B | 8.32 | 7.27 | 51.70 | 21.59 | 30.11 | 14.32 | 247.12 | 3.86 | 5.47 | 14.18 | 23.51 | 241.71 | 265.22 |
| S7A1S | 13.23 | 2.43 | 50.09 | 18.26 | 31.83 | 15.83 | 296.85 | 1.28 | 2.12 | 40.93 | 44.33 | 420.42 | 464.75 |
| S7A2B | 1.09 | 1.68 | 19.33 | 16.00 | 3.33 | 14.32 | 380.00 | 2.93 | 3.50 | 31.51 | 37.94 | 205.70 | 243.64 |
| S7A2M | 1.27 | 1.79 | 17.61 | 11.48 | 6.13 | 9.70 | 327.10 | 3.07 | 17.69 | 22.88 | 43.64 | 385.64 | 429.27 |
| S7A2S | 3.11 | 3.83 | 29.76 | 9.55 | 20.22 | 5.72 | 289.40 | 3.03 | 13.20 | 9.81 | 26.03 | 389.09 | 415.12 |
| S7B1B | 5.25 | 2.00 | 21.38 | 9.55 | 11.83 | 7.55 | 287.41 | 3.64 | 15.50 | 25.26 | 44.40 | 365.15 | 409.56 |
| S7B1S | 3.48 | 2.54 | 20.95 | 9.33 | 11.61 | 6.80 | 345.92 | 1.99 | 7.38 | 31.83 | 41.20 | 277.80 | 318.99 |
| S7B2B | 3.05 | 2.54 | 22.56 | 12.45 | 10.11 | 9.91 | 315.26 | 4.17 | 20.89 | <D.L | 25.06 | 201.23 | 226.29 |
| S7B2M | 3.21 | 1.79 | 39.66 | 15.25 | 24.41 | 13.46 | 420.99 | 2.94 | 27.71 | 10.80 | 41.45 | 225.52 | 266.97 |
| S7B2S | 1.51 | 2.54 | 13.31 | 10.19 | 3.12 | 7.66 | 239.15 | 1.49 | 10.99 | 27.52 | 39.99 | 171.74 | 211.72 |
| S7C1B | 14.25 | 21.14 | 70.41 | 25.25 | 45.16 | 4.11 | 357.87 | 3.94 | 5.80 | 7.02 | 16.75 | 240.99 | 257.74 |
| S7C1S | 15.41 | 23.08 | 73.53 | 32.45 | 41.08 | 9.38 | 327.69 | 1.69 | 2.16 | 37.34 | 41.19 | 244.06 | 285.26 |
| S7C2B | 0.98 | 1.68 | 18.04 | 12.02 | 6.02 | 10.34 | 252.53 | 3.96 | 23.35 | 19.19 | 46.50 | 217.45 | 263.95 |
| S7C2M | 2.32 | 1.03 | 12.67 | 10.62 | 2.04 | 9.59 | 300.06 | 3.28 | 13.08 | 8.16 | 24.52 | 245.48 | 270.00 |
| S7C2S | 1.65 | 1.68 | 40.95 | 13.85 | 27.10 | 12.17 | 284.67 | 1.06 | 2.59 | 12.98 | 16.63 | 204.24 | 220.87 |

^*^D.L = detection limit
