# Supplementary material for: Spatiotemporal Distribution and Assemblages of Planktonic Fungi in the Coastal Waters of the Bohai Sea
Source: Front Microbiol. 2018 Mar 28;9:584. doi: 10.3389/fmicb.2018.00584 (PMC5882831; doi:10.3389/fmicb.2018.00584)
Supplement: Supplementary file 2 [file Table_2.docx]

Supplementary Material

Spatiotemporal Distribution and Assemblages of Planktonic Fungi in the Coastal Waters of the Bohai Sea

**Yaqiong Wang^1, 2, †^, Biswarup Sen^1, †^, Yaodong He^1^, Ningdong Xie^1, 3^, Guangyi Wang^1, 4, *^**

^1^ Center for Marine Environmental Ecology, School of Environment Science and Engineering, Tianjin University, Tianjin, China

^2^ School of Ecology, Environment and Resources, Qinghai University for Nationalities, Xining, China

^3^ Duke Marine Laboratory, Nicholas School of the Environment, Duke University, Durham, NC, United States

^4^ Key Laboratory of Systems Bioengineering (Ministry of Education), Tianjin University, Tianjin, China

*** Correspondence:** Corresponding Author: [gywang@tju.edu.cn](mailto:gywang@tju.edu.cn)

^†^These authors contributed equally to this work

**Supplementary Table S2 Variations in α-diversity (Shannon index) across different seasons, sections, and depths in the Qinhuangdao coastal waters.**

| **Season** | **Section** | **Depth^*^** | **Shannon** |
| --- | --- | --- | --- |
| November | A1 | B | 3.56±0.02 |
|  |  | S | 4.42±0.02 |
|  | A2 | B | 3.83±0.03 |
|  |  | M | 3.61±0.01 |
|  |  | S | 4.16±0.02 |
|  | B1 | B | 4.20±0.02 |
|  |  | S | 3.79±0.02 |
|  | B2 | B | 3.60±0.02 |
|  |  | M | 3.69±0.01 |
|  |  | S | 3.73±0.02 |
|  | C1 | B | 3.00±0.02 |
|  |  | S | 1.19±0.03 |
|  | C2 | B | 2.79±0.02 |
|  |  | M | 2.53±0.03 |
|  |  | S | 1.87±0.03 |
| April | A1 | B | 2.82±0.03 |
|  |  | S | 3.08±0.03 |
|  | A2 | B | 4.41±0.02 |
|  |  | M | 3.98±0.02 |
|  |  | S | 3.04±0.03 |
|  | A3 | B | 1.03±0.03 |
|  |  | M | 3.98±0.02 |
|  |  | S | 3.79±0.02 |
|  | B1 | B | 3.54±0.02 |
|  |  | S | 3.39±0.02 |
|  | B2 | B | 3.12±0.02 |
|  |  | M | 3.17±0.02 |
|  |  | S | 2.35±0.03 |
|  | B3 | B | 2.09±0.03 |
|  |  | M | 2.21±0.02 |
|  |  | S | 2.27±0.03 |
|  | C1 | B | 3.51±0.03 |
|  |  | S | 2.55±0.03 |
|  | C2 | B | 3.99±0.02 |
|  |  | M | 3.28±0.03 |
|  |  | S | 3.80±0.03 |
|  | C3 | B | 3.39±0.03 |
|  |  | M | 3.46±0.02 |
|  |  | S | 3.32±0.03 |
| July | A1 | B | 3.26±0.02 |
|  |  | S | 2.86±0.02 |
|  | A2 | B | 3.29±0.03 |
|  |  | M | 2.78±0.02 |
|  |  | S | 2.65±0.03 |
|  | B1 | B | 1.56±0.02 |
|  |  | S | 2.22±0.03 |
|  | B2 | B | 3.63±0.03 |
|  |  | M | 2.66±0.03 |
|  |  | S | 1.67±0.03 |
|  | C1 | B | 3.33±0.02 |
|  |  | S | 3.58±0.02 |
|  | C2 | B | 4.11±0.01 |
|  |  | M | 2.88±0.02 |
|  |  | S | 3.07±0.03 |

^*^B=bottom water sample, S=surface water sample, M=middle water sample
