# Supplementary material for: Spatiotemporal Distribution and Assemblages of Planktonic Fungi in the Coastal Waters of the Bohai Sea
Source: Front Microbiol. 2018 Mar 28;9:584. doi: 10.3389/fmicb.2018.00584 (PMC5882831; doi:10.3389/fmicb.2018.00584)

Supplementary Material

Spatiotemporal Distribution and Assemblages of Planktonic Fungi in the Coastal Waters of the Bohai Sea

Yaqiong Wang[1, 2, †], Biswarup Sen[1, †], Yaodong He[1], Ningdong Xie[1, 3], Guangyi Wang[1, 4, *]

[1] Center for Marine Environmental Ecology, School of Environment Science and Engineering, Tianjin University, Tianjin, China
[2] School of Ecology, Environment and Resources, Qinghai University for Nationalities, Xining, China
[3] Duke Marine Laboratory, Nicholas School of the Environment, Duke University, Durham, NC, United States
[4] Key Laboratory of Systems Bioengineering (Ministry of Education), Tianjin University, Tianjin, China

*** Correspondence:** Corresponding Author: gywang@tju.edu.cn
[†]These authors contributed equally to this work

Supplementary Figure S1 Frequency of dominant planktonic fungal orders in individual plankton samples collected from Qinhuangdao coastal area (>1% of sequences in libraries).

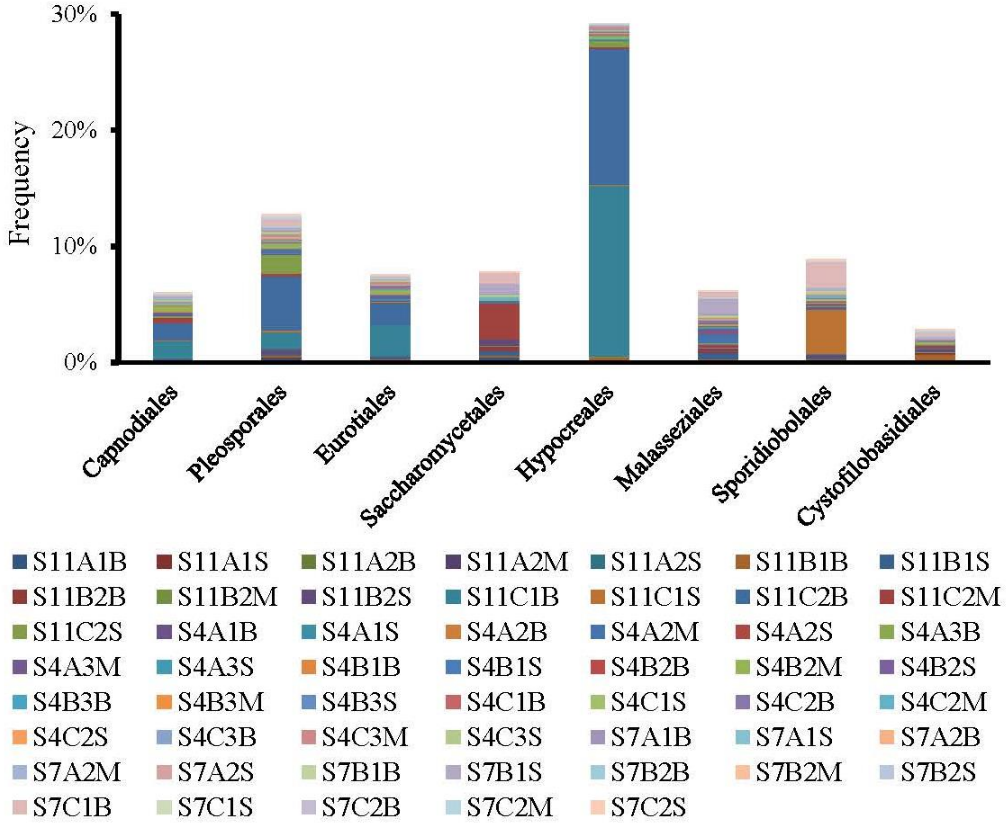

Supplement: Supplementary file 3 [file Image_1.pdf]
